# Supplementary material for: Developmental single-cell atlas of coronary vessel growth and cardiomyocyte interaction in zebrafish
Source: Development. 2026 Jan 22;153(2):dev205065. doi: 10.1242/dev.205065 (PMC12863305; doi:10.1242/dev.205065)
Supplement: Supplementary information [file develop-153-205065-s1.pdf]

Supplementary Figure 1

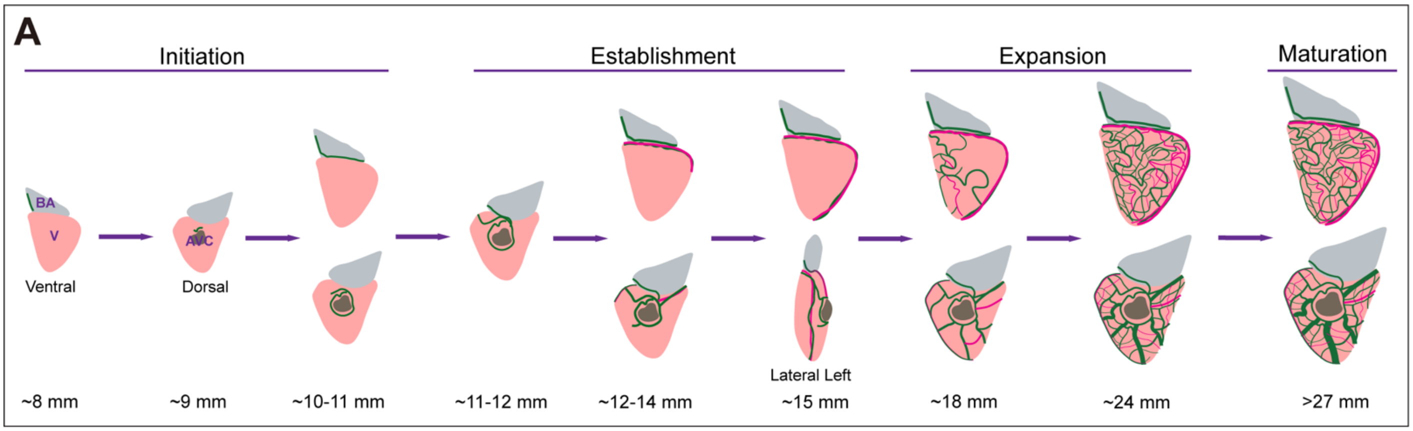

Fig. S1. Schematic diagram of coronary network development (Related to Figure 1)

(**A**) Schematic representation of coronary vessel development from the initiation to maturation stages corresponding to zebrafish body length.

Development • Supplementary information

Supplementary Figure 2

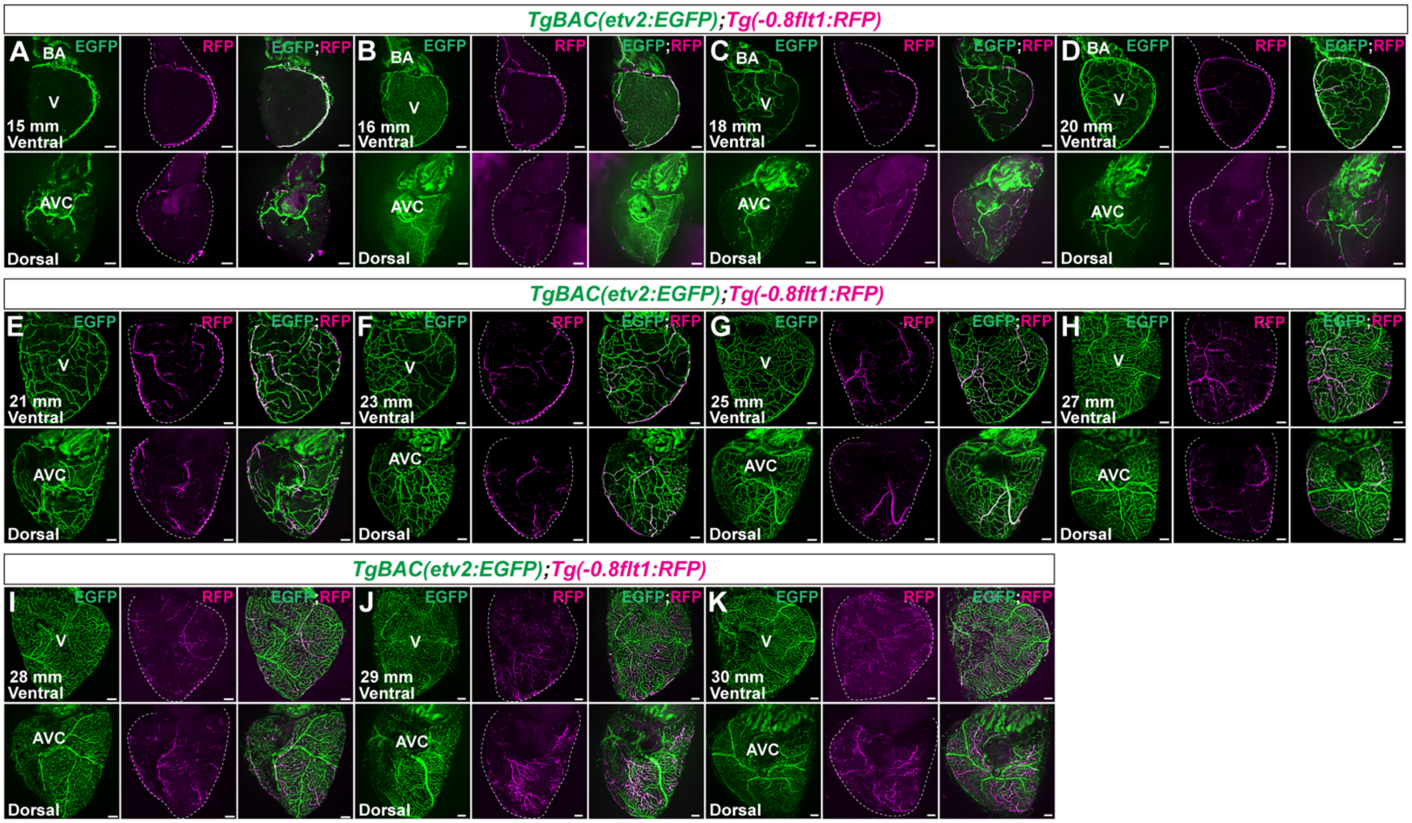

Fig. S2. Characterization of coronary development from the expansion to maturation stages (Related to Figure 1)

(**A-K**) Wholemount images of *TgBAC(etv2:EGFP)*; *Tg(-0.8flt1:RFP)* ventricles from 15- to 20-mm (expansion I stage) (**A-D**), 21- to 27-mm (expansion II stage) (**E-H**), and 28- to 30-mm (maturation stage) (**I-K**) long zebrafish. Scale bars: 100 µm.

Development • Supplementary information

Supplementary Figure 3

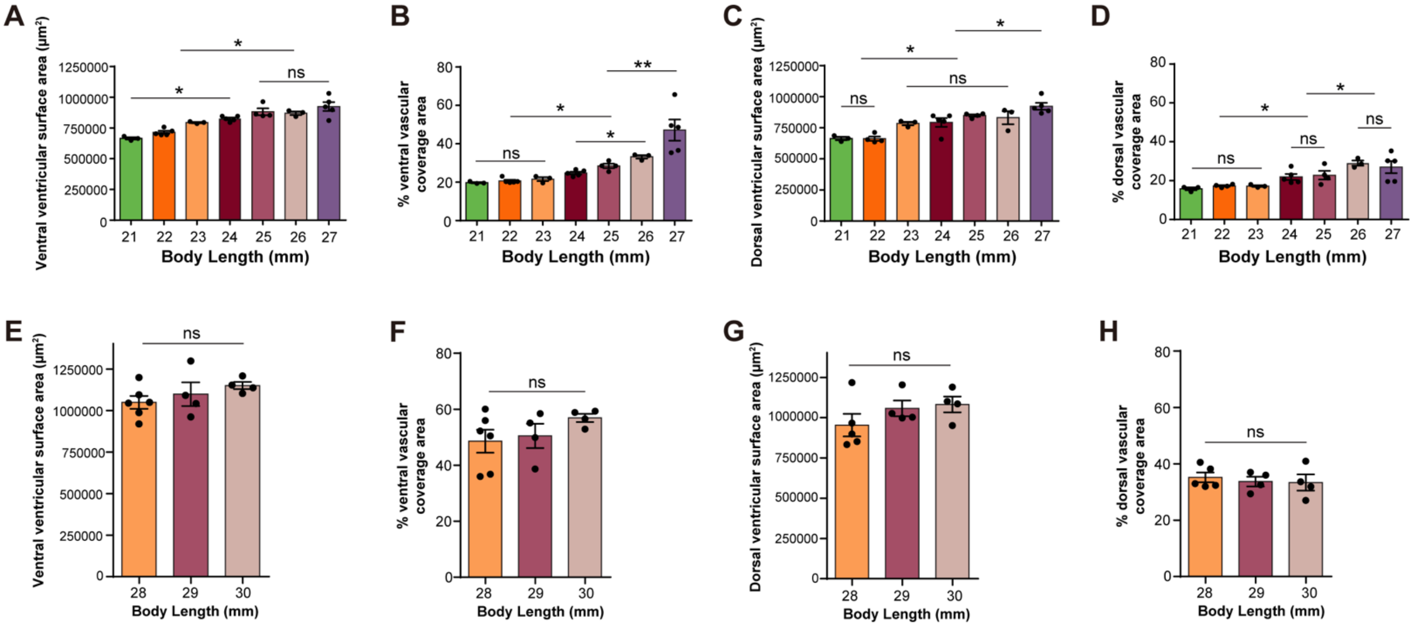

Fig. S3. Coronary network growth quantification during the expansion II and maturation stages (Related to Figure 1)

(**A-D**) Quantification of ventral (**A**) and dorsal (**C**) ventricular surface areas, percentage of ventral (**B**) and dorsal (**D**) vessel coverage in 21- to 27-mm-long zebrafish (expansion II stage).

(**E-H**) Quantification of ventral (**E**) and dorsal (**G**) ventricular surface areas, percentage of ventral (**F**) and dorsal (**H**) vessel coverage in 28- to 30-mm-long zebrafish (maturation stage).

Data in graphs expressed as mean ± SEM. ns, no significant difference, $*p < 0.05$, $**p < 0.01$.

Supplementary Figure 4

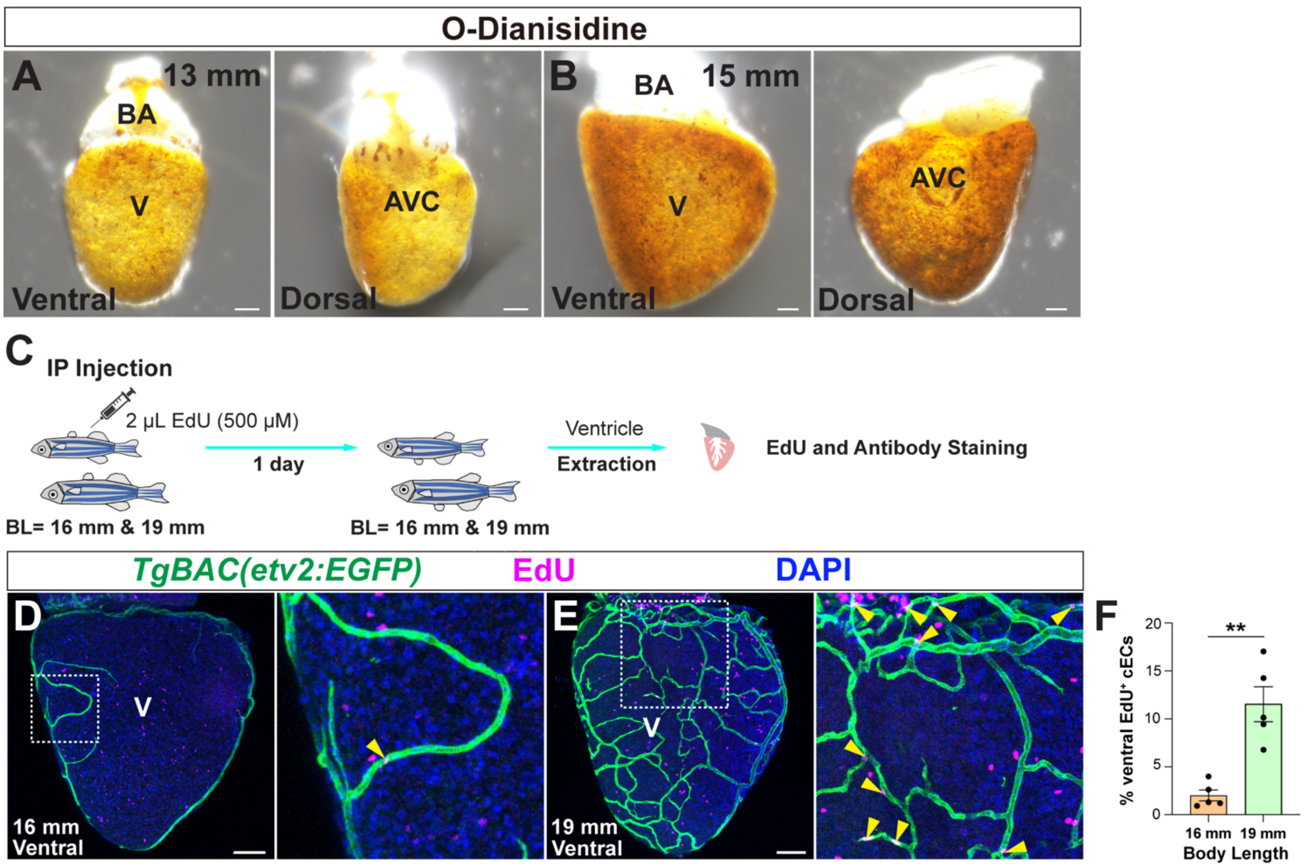

Fig. S4. O-Dianisidine and EdU stainings (Related to Figure 1)

(**A**) Wholemount ventricles from 13- and 15-mm-long zebrafish stained with O-Dianisidine.

(**C**) Schematic diagram of EdU administration and antibody staining. BL, body length.

(**D-E**) Wholemount images of *TgBAC(etv2:EGFP)* ventricles from 16- (**D**) and 19- (**E**) mm-long zebrafish stained for EGFP (coronary ECs, green), EdU (proliferating cells, magenta) and DNA (blue). Yellow arrowheads point to EdU$^+$ cECs.

(**F**) Percentage ventral EdU$^+$ cECs in 16- and 19-mm-long zebrafish.

Data in graphs expressed as mean ± SEM. $**p < 0.01$. Scale bars: 100 μm.

Supplementary Figure 5

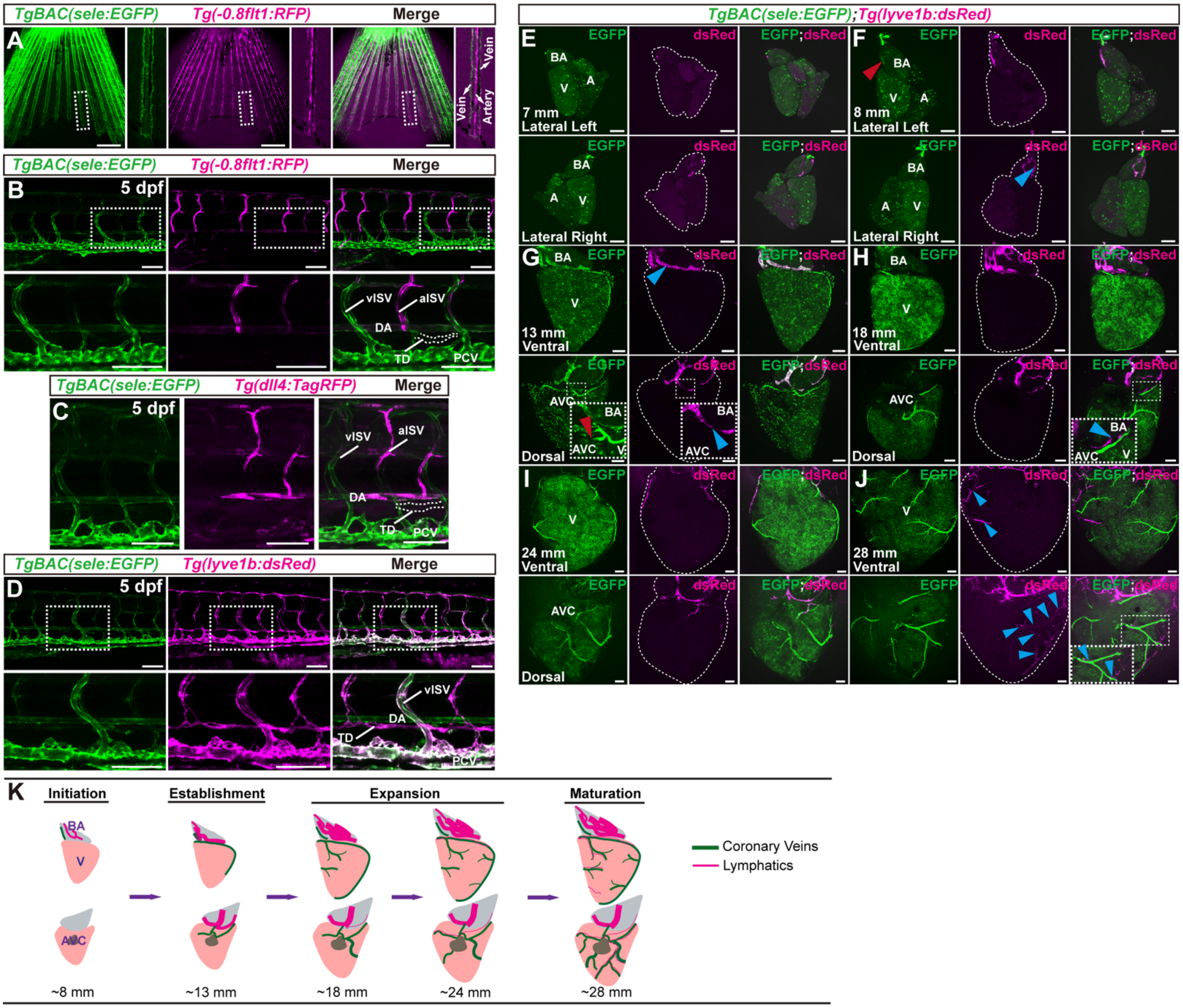

Fig. S5. *TgBAC(sele:EGFP)* expression (Related to Fig. 2)

(**A**) Images of an adult *TgBAC(sele:EGFP)*; *Tg(-0.8flt1:RFP)* zebrafish fin. Insets show high-magnification images of a fin ray. EGFP^{+}/RFP^{low} signal is detected in veins and EGFP^{+}/RFP^{high} labels arterial vessels.

(**B-D**) Images of zebrafish trunk vasculature in *TgBAC(sele:EGFP)*; *Tg(-0.8flt1:RFP)* (**B**), *TgBAC(sele:EGFP)*; *Tg(dll4:TagRFP)* (**C**) and *TgBAC(sele:EGFP)*; *Tg(lyve1b:dsRed* (**D**) at 5 days post-fertilization (dpf). aISV, arterial intersegmental vessels; vISV, venous intersegmental vessels; DA, dorsal aorta; TD, thoracic duct; PCV, posterior cardinal vein.

(**E,F**) Wholemount images of *TgBAC(sele:EGFP)*; *Tg(lyve1b:dsRed)* hearts from 7- (**E**) and 8- (**F**) mm-long zebrafish. Red arrowhead points to a *sele*:EGFP^{+} vessel, blue arrowhead points to *lyve1b*:dsRed^{+} lymphatic vessels on the BA.

(**G,H**) Wholemount images of *TgBAC(sele:EGFP)*; *Tg(lyve1b:dsRed)* ventricles from 13- (**G**) and 18- (**H**) mm-long zebrafish. Red arrowhead points to *sele*:EGFP^{+} vessels extending from the AVC, blue arrowheads point to *lyve1b*:dsRed^{+} lymphatic vessels in the junction between the BA and the ventricular base.

(**I,J**) Wholemount images of *TgBAC(sele:EGFP)*; *Tg(lyve1b:dsRed)* ventricles from 24- (**I**) and 28- (**J**) mm-long zebrafish. Blue arrowheads point to *lyve1b*:dsRed^{+} lymphatic vessels on the ventricular surface.

(**K**) Schematic diagram of coronary vein and lymphatic development.

Scale bars: 1000 μm (**A**), 500 μm (**B-D**), 100 μm (**E-K**).

Supplementary Figure 6

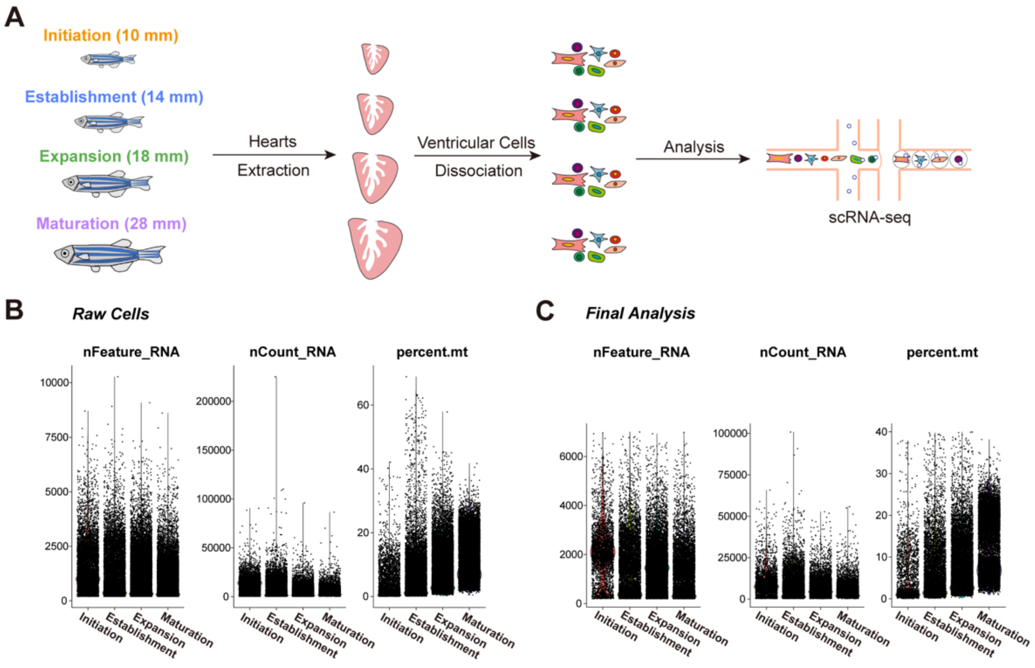

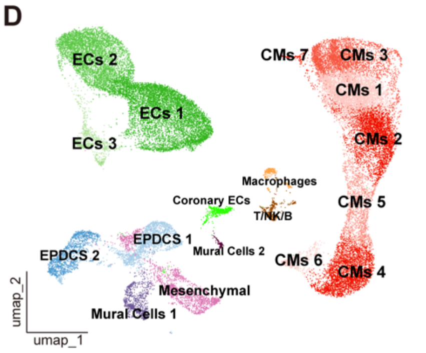

E

Cell Number				
**Cell Cluster**	**Initiation**	**Establishment**	**Expansion**	**Maturation**
CMs 1	104	774	1276	2338
CMs 2	154	908	1111	1688
CMs 3	50	754	829	1856
CMs 4	584	1286	1190	194
CMs 5	106	275	609	507
CMs 6	55	125	174	61
CMs 7	20	134	1	0
ECs 1	409	1180	3385	2097
ECs 2	619	778	2998	486
ECs 3	197	174	386	145
Coronary ECs	5	4	148	322
EPDCs 1	163	229	1035	675
EPDCs 2	181	201	576	345
Mural Cells 1	345	78	423	66
Mural Cells 2	0	1	58	82
Mesenchymal	111	266	1036	473
Macrophages	30	65	89	117
T/NK/B	58	102	136	117
**Total**	**3191**	**7334**	**15460**	**11569**

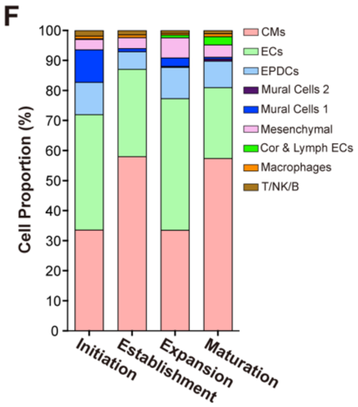

Fig. S6. scRNA-seq analysis during heart development (Related to Figure 3)

(**A**) Experimental workflow of ventricular cell dissociation from zebrafish hearts at four different stages of coronary development for scRNA-seq analysis.

(**B,C**) Quality control metrics of scRNA-seq data in raw cells (**B**) and final analysis (**C**).

(**D**) Combined UMAP plot visualizing eighteen cell clusters identified using aggregated data of four coronary developmental stages.

(**E,F**) Cell number per cluster (**E**) and cell proportion percentage (**F**) at each stage.

Development • Supplementary information

Supplementary Figure 7

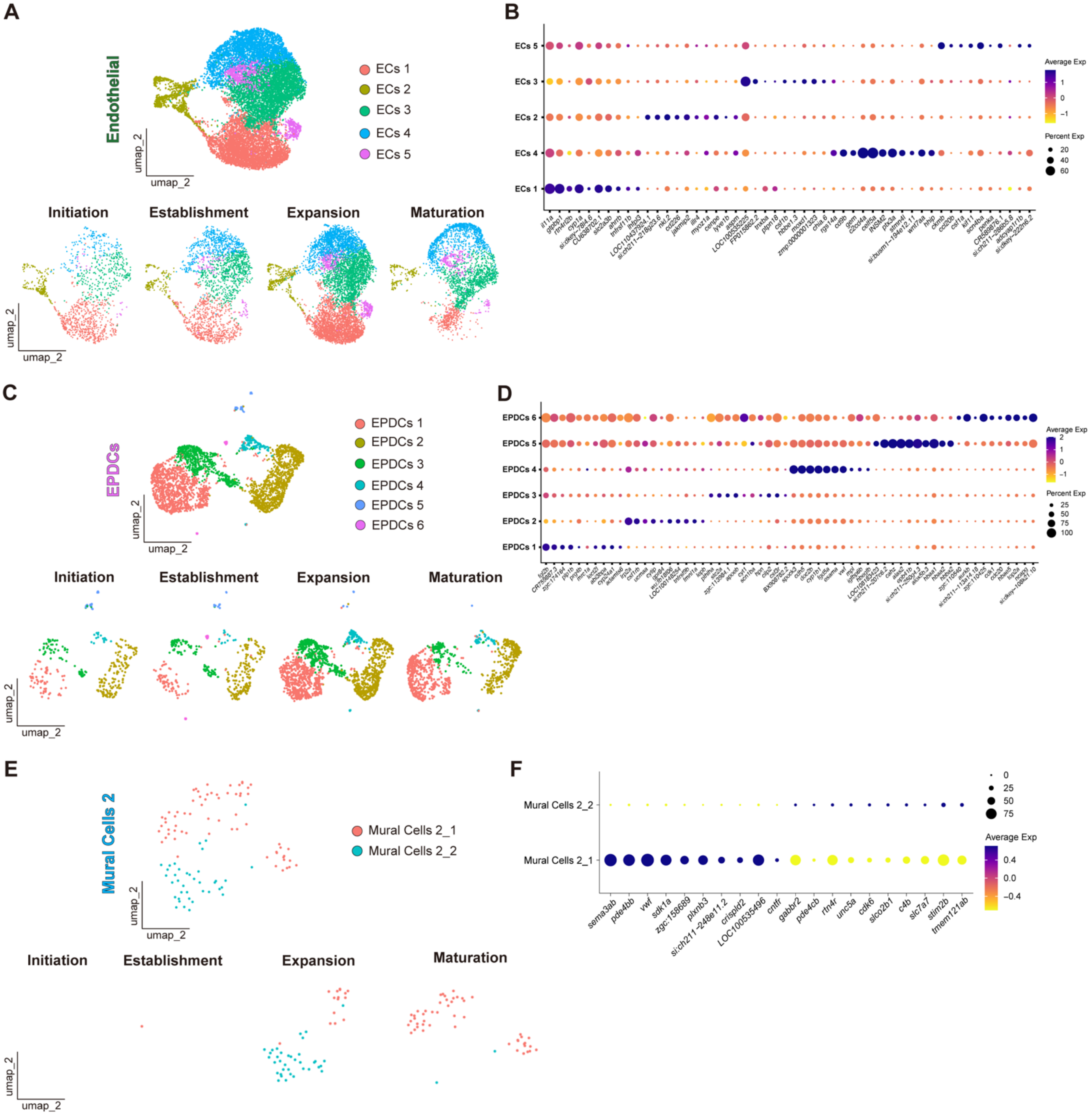

Fig. S7. Secondary subclustering of ECs, EPDCs and mural cells 2 (Related to Figure 3) (**A**) Combined UMAP plot of ECs showing five subclusters identified using aggregated data from all stages analysed and followed by split UMAP plots showing these subclusters at each stage. (**B**) Dot plot showing average expression and abundance of top ten marker genes in ECs.

(**C**) Combined UMAP plots of EPDCs showing six subclusters identified using aggregated data from all stages analysed and followed by split UMAP plots showing these subclusters at each stage.

(**D**) Dot plot showing average expression and abundance of top ten marker genes in EPDCs.

(**E**) Combined UMAP plots of Mural Cells 2 showing two subclusters identified using aggregated data from all stages analysed and followed by split UMAP plots showing these subclusters at each stage.

(**F**) Dot plot showing average expression and abundance of top ten marker genes in Mural Cells 2.

Supplementary Figure 8

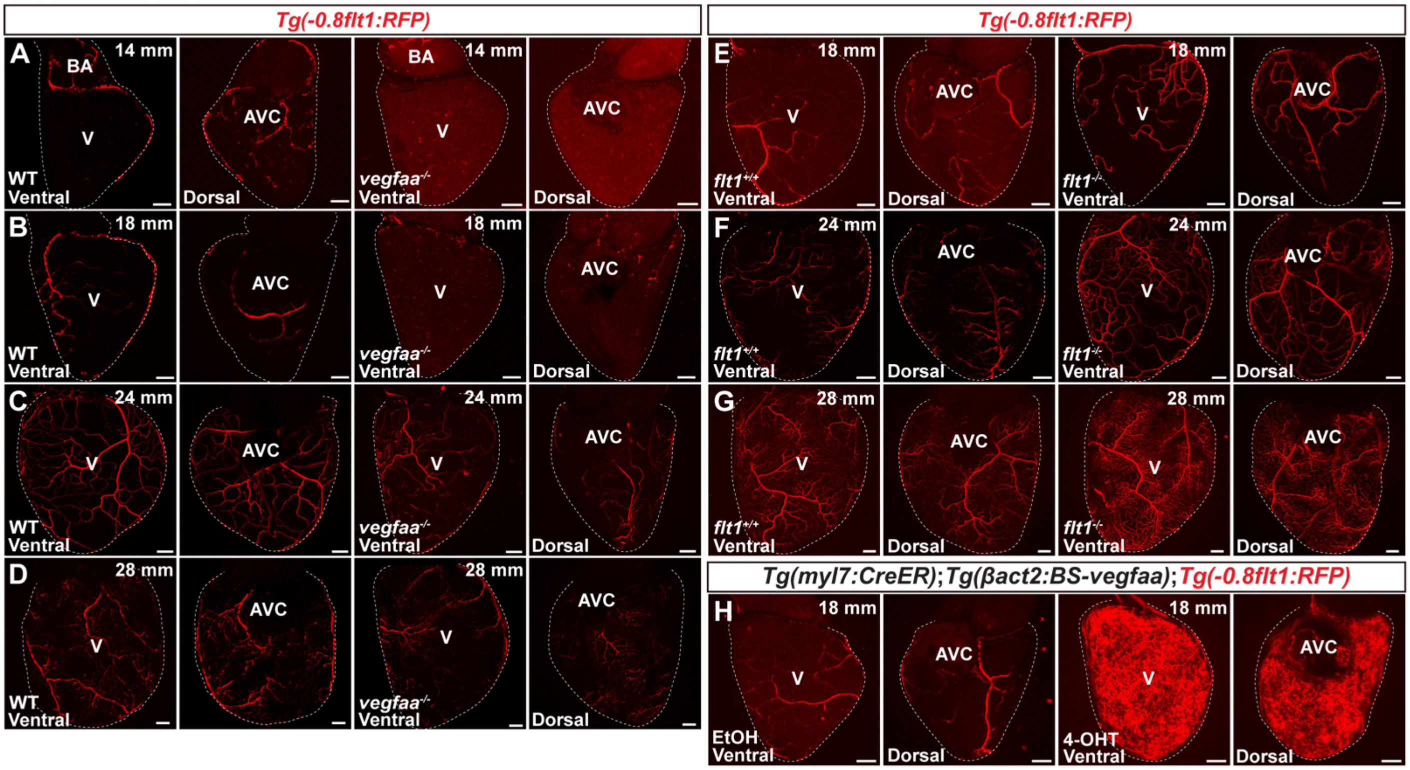

Fig. S8. Manipulation of Vegfa signaling alters *-0.8flt1*:RFP expression (Related to Fig. 4 and 5)

(**A-D**) Wholemount images of *Tg(-0.8flt1:RFP)* ventricles from 14- (**A**), 18- (**B**), 24- (**C**) and 28- (**D**) mm-long WT and rescued *vegfaa*$^{-/-}$.

(**E-G**) Wholemount images of *Tg(-0.8flt1:RFP)* ventricles from 18- (**E**), 24- (**F**) and 28- (**G**) mm-long *flt1*$^{+/+}$ and *flt1*$^{-/-}$.

(**H**) Wholemount images of *Tg(myl7:CreER)*; *Tg(βact2:BS-vegfaa)*; *Tg(-0.8flt1:RFP)* ventricles from 18-mm-long control and tamoxifen (4-OHT) treated (*vegfaa*^{OE}) fish.

Scale bars: 100 µm.

Development • Supplementary information

Supplementary Figure 9

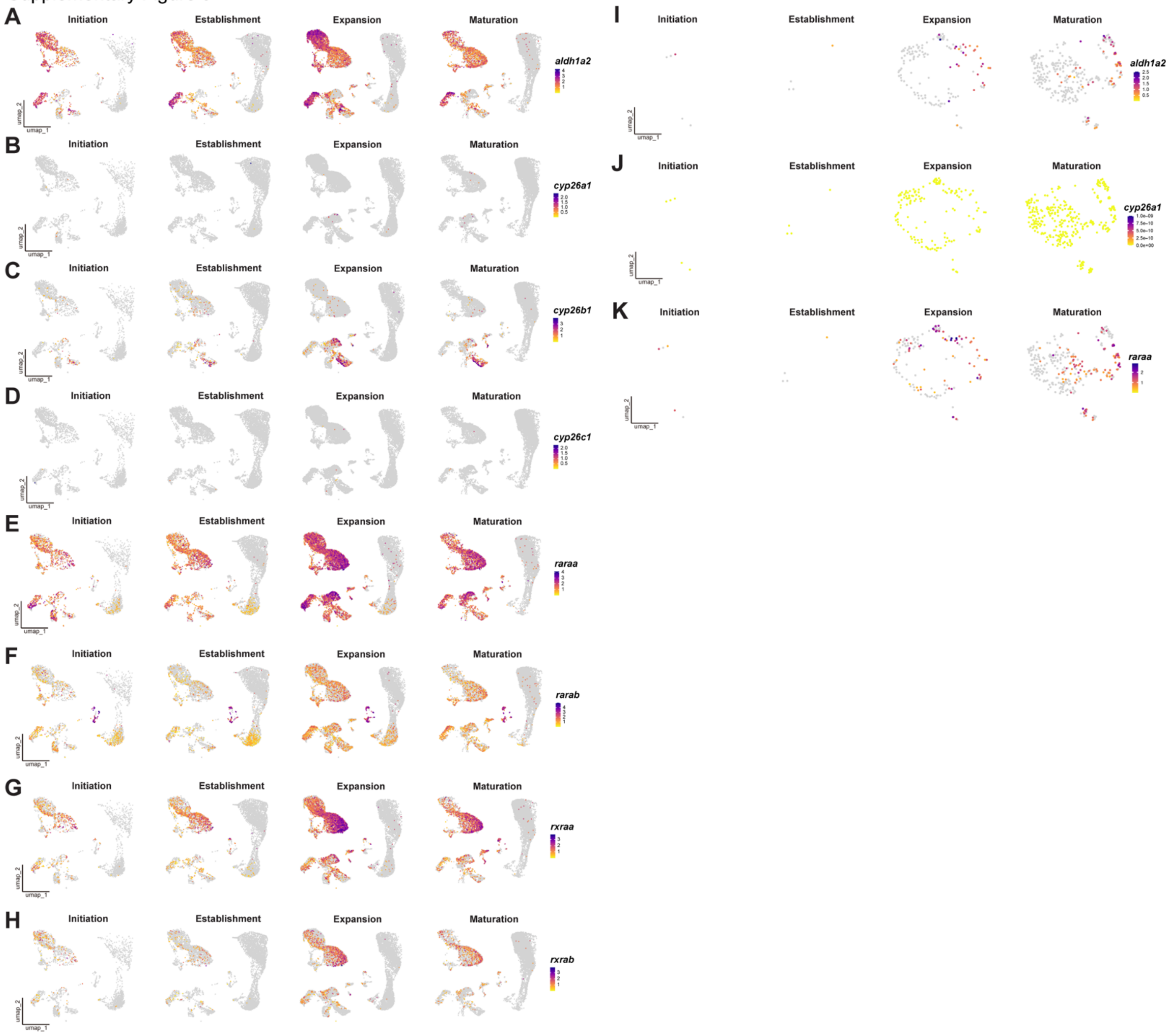

Fig. S9. Expression analysis of genes encoding components of retinoic acid (RA) signaling across all cell types during coronary development (Related to Figure 4)

(**A-H**) UMAP plots showing *aldh1a2* (**A**), *cyp26a1* (**B**), *cyp26b1* (**C**), *cyp26c1* (**D**), *raraa* (**E**), *rarab* (**F**), *rxraa* (**G**) and *rxrab* (**H**) expression in all ventricular cells during different stages of coronary development.

(**I-K**) UMAP plots showing *aldh1a2* (**I**), *cyp26a1* (**J**) and *raraa* (**K**) expression in coronary ECs during different stages of coronary development.

Development • Supplementary information

Supplementary Figure 10

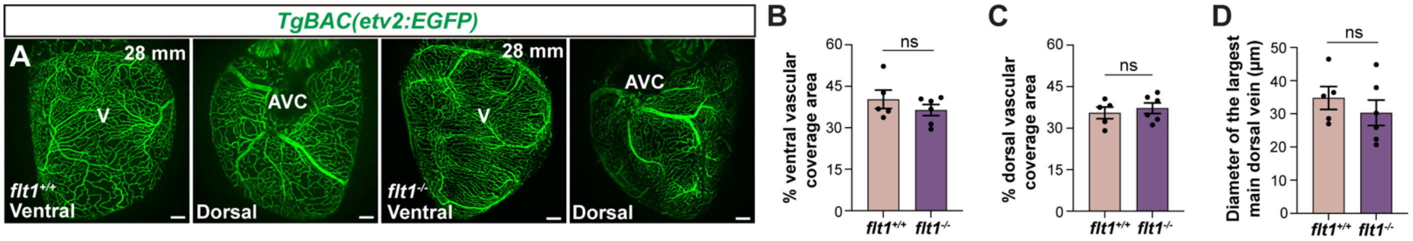

Fig. S10. *flt1* mutant phenotypes at maturation stage (Related to Figure 5)

(**A**) Wholemount images of *TgBAC(etv2:EGFP)* ventricles from 28-mm-long *flt1*$^{+/+}$ and *flt1*$^{-/-}$.

(**B-D**) Quantification of ventral (**B**) and dorsal (**C**) percentage of vessel coverage, and diameter of the largest main dorsal coronary vein (**D**) in 28-mm-long *flt1*$^{+/+}$ and *flt1*$^{-/-}$.

Data in graphs expressed as mean ± SEM. ns, no significant difference. Scale bars: 100 μm.

Supplementary Figure 11

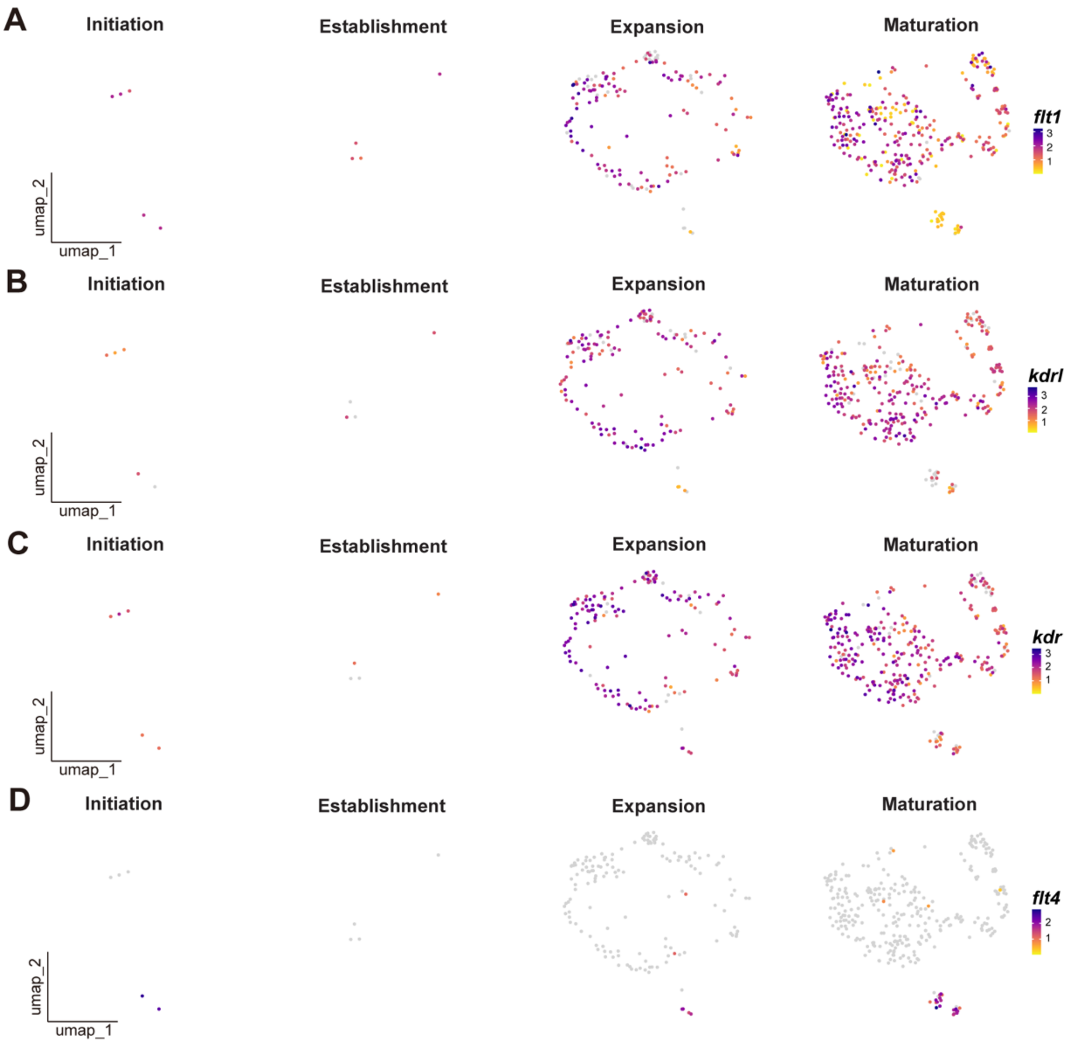

Fig. S11. Expression analysis of genes encoding Vegf receptors in coronary ECs during coronary development (Related to Figure 4 and 5)

(**A-D**) UMAP plots showing *flt1* (**A**), *kdrl* (**B**), *kdr* (**C**) and *flt4* (**D**) expression in coronary ECs during different stages of coronary development.

Development • Supplementary information

Supplementary Figure 12

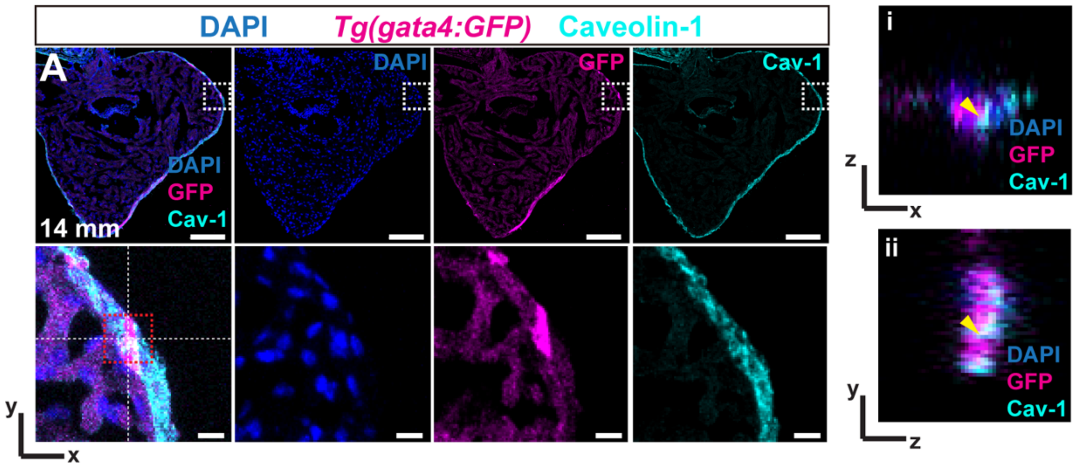

Fig. S12. Non-cardiomyocyte *gata4*:GFP^{+} expression in 14 mm long zebrafish (Related to Fig. 6)

(**A**) *Tg(gata4:GFP)* ventricular section from a 14-mm-long zebrafish stained for epicardium (Caveolin-1, cyan), *gata4*:GFP (magenta) and DNA (blue). Orthogonal views in xz (**i**) and yz (**ii**) axes. Yellow arrowheads point to co-localization of DAPI^{+}, GFP^{+} and Cav-1^{+} signals.

Development • Supplementary information

Supplementary Figure 13

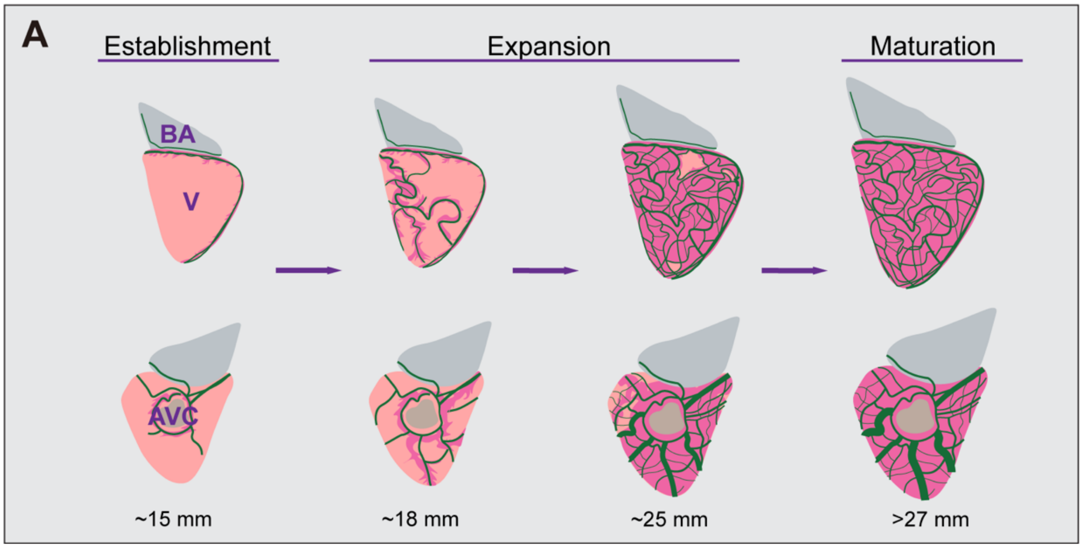

Fig. S13. Schematic diagram of CM growth and expansion (Related to Fig. 6)

(**A**) Schematic representation showing cortical CM growth and expansion in coordination with coronary vessels.

Development • Supplementary information

Supplementary Figure 14

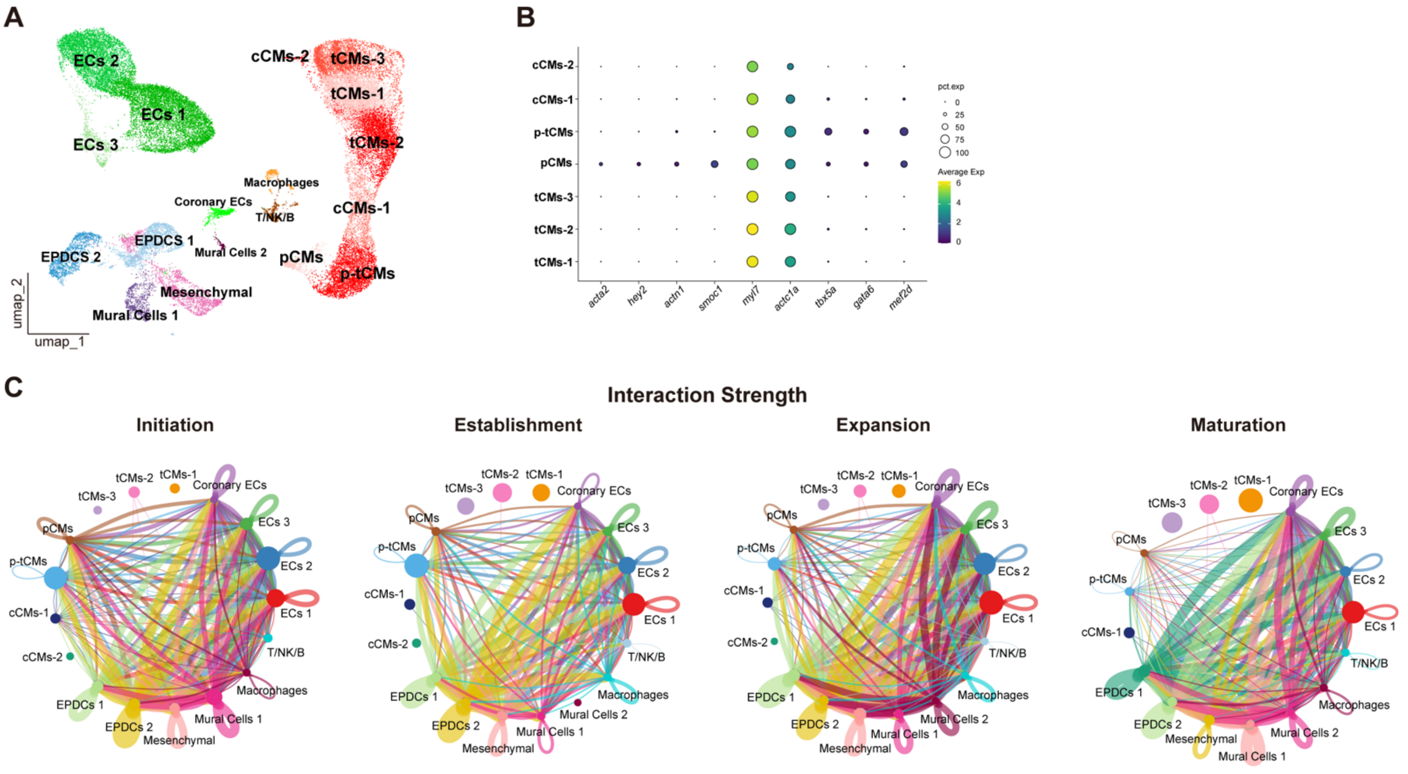

Fig. S14. Identification of CM clusters and analysis of cell-cell interaction strength among all ventricular components (Related to Fig. 7)

(**A,B**) Combined UMAP plot visualizing eighteen cell clusters, including seven CM subtypes identified using aggregated data of four coronary developmental stages (**A**) and dot plot showing average expression and abundance of selected CM marker genes corresponding to CM subtypes (**B**). pCMs, primordial CMs; p-tCMs, primordial-trabecular CMs; tCMs, trabecular CMs; cCMs, cortical CMs.

(**C**) Circle plots showing differential cell-cell communication networks between all ventricular components during coronary development. Each node in the circle plot represents a cell cluster, with the node size being proportional to cell number. Edges connect nodes, indicating communication between cell clusters. Edge width represents communication strength.

Development • Supplementary information

Supplementary Figure 15

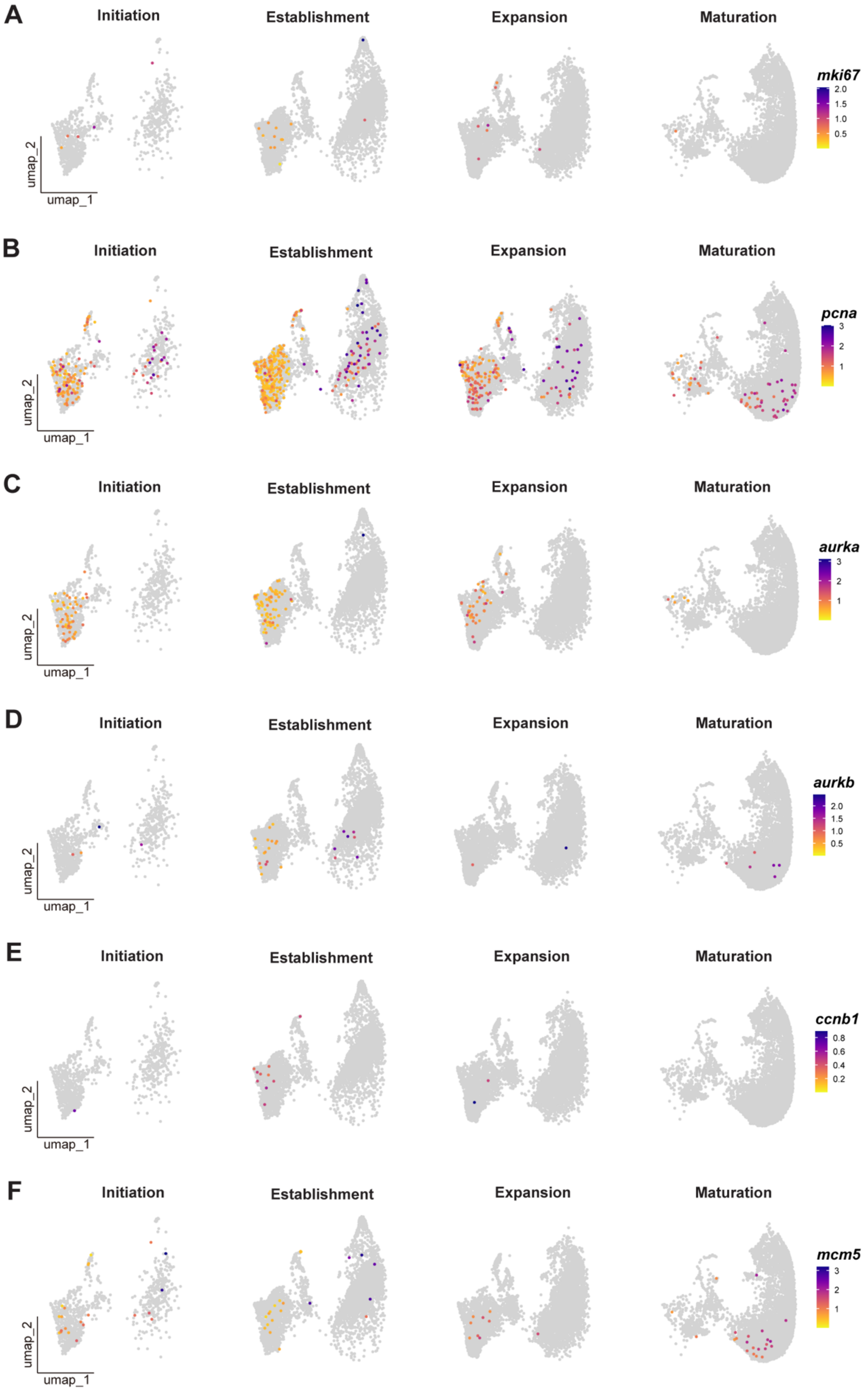

Development • Supplementary information

Fig. S15. Expression analysis of genes encoding cell-cycle regulators in CMs during coronary development (Related to Fig. 7)

(**A-F**) UMAP plots showing *mki67* (**A**), *pcna* (**B**), *aurka* (**C**), *aurkb* (**D**), *ccnb1* (**E**) and *mcm5* (**F**) expression in CMs during different stages of coronary development.

Table S1. Summary of scRNA-seq QC Metrics

Available for download at
https://journals.biologists.com/dev/article-lookup/doi/10.1242/dev.205065#supplementary-data

Table S2. Number of cells retained

Available for download at
https://journals.biologists.com/dev/article-lookup/doi/10.1242/dev.205065#supplementary-data

Table S3. All markers at each cluster

Available for download at
https://journals.biologists.com/dev/article-lookup/doi/10.1242/dev.205065#supplementary-data

Table S4. Initiation all interactions

Available for download at
https://journals.biologists.com/dev/article-lookup/doi/10.1242/dev.205065#supplementary-data

Development • Supplementary information

Table S5. Establishment all interactions

Available for download at
https://journals.biologists.com/dev/article-lookup/doi/10.1242/dev.205065#supplementary-data

Table S6. Expansion all interactions

Available for download at
https://journals.biologists.com/dev/article-lookup/doi/10.1242/dev.205065#supplementary-data

Table S7. Maturation all interactions

Available for download at
https://journals.biologists.com/dev/article-lookup/doi/10.1242/dev.205065#supplementary-data

Development • Supplementary information
